# Anatomical study: comparing the human, sheep and pig knee meniscus

**DOI:** 10.1186/s40634-016-0071-3

**Published:** 2016-12-07

**Authors:** Talal Takroni, Leila Laouar, Adetola Adesida, Janet A. W. Elliott, Nadr M. Jomha

**Affiliations:** 1 Department of Surgery, Laboratory of Orthopaedic Research, University of Alberta, Edmonton, Canada; 2 Department of Surgery, Laboratory of Stem Cell Biology and Orthopaedic Tissue Engineering, University of Alberta, Edmonton, Canada; 3 Department of Chemical and Materials Engineering, University of Alberta, Edmonton, Canada; 4 Department of Laboratory Medicine and Pathology, University of Alberta, Edmonton, Canada; 5 Rabigh Faculty of Medicine, King Abdulaziz University, Jeddah, Kingdom of Saudi Arabia

## Abstract

**Background:**

Animal models are commonly used in investigating new treatment options for knee joint injuries including injuries to the meniscus. The reliability and applicability of these models to replicate findings in humans depends on determining the most suitable animal proxy. Therefore, this study was designed to compare the wet weight, volume and dimensions of the human meniscus with two commonly used animal models: sheep and pig.

**Methods:**

Human menisci (*n* = 6 pairs) were obtained from the knee joints of cadaveric male donors. Sheep menisci (*n* = 6 pairs) and pig menisci (*n* = 22 pairs) were obtained from the stifle joints of adult sheep and pigs. Meniscal wet weight, volume and dimensions of the body were measured and compared among the species. Anatomical dimensions included circumference, width, peripheral height, articular height and superior articular length.

**Results:**

The circumference of human menisci (lateral: 84.0 mm, medial: 88.7 mm) was significantly longer than that of sheep (lateral: 50.0 mm, medial: 55.5 mm) and pig (lateral: 66.8 mm, medial: 64.9 mm). The majority of the remaining dimensions of the medial and all of the remaining dimensions of the lateral menisci in sheep showed no statistical difference in comparison to the human menisci. The meniscal weight in pig was significantly larger (lateral: 6.4 g, medial: 5.0 g) than the human (lateral: 4.9 g, medial: 4.4 g) and sheep (lateral: 2.5 g, medial: 2.2 g). Porcine meniscal volume (lateral: 6.5 ml, medial: 5.1 ml) was also larger than the human (lateral: 5.0 ml, medial: 4.5 ml) and sheep (lateral: 2.3 ml, medial: 2.2 ml) menisci. The dimensions measured in the pig meniscus were generally larger than human menisci with statistically significant differences in most categories.

**Conclusion:**

Sheep meniscal dimensions more closely matched human meniscal dimensions than the pig meniscal dimensions. This information may help guide the choice of an animal proxy in meniscal research.

## Background

The meniscus holds considerable clinical significance in the human knee and the anatomical features demonstrate propensity for injury (Makris et al. 2011). The tendency of human menisci to injury has motivated scientists to look for effective options to treat patients with meniscal injury. However, before a new treatment option can be translated into clinical practice in humans, it has to go through a testing phase in an animal model. The menisci of all mammals, regardless of the walking style or size have the same semilunar shape (Parsons 1899). Various animal models, especially large domestic quadrupeds, have been utilized in the development of new and successful treatment options for meniscal injury (Deponti et al. 2015). However, the ability to predict responses in human menisci has not been determined for any model.

Examples of animal models that have been utilized previously in meniscal research include dog, pig, sheep, goat, cow and rabbit (Aspden et al. 1985; Chevrier et al. 2009; Deponti et al. 2015; Ghadially et al. 1978; Ghadially et al. 1986; Krause et al. 1976). Despite the contribution of these models to the overall understanding of meniscus biology, no single animal model has been identified as the most suitable for human meniscal research (Arnoczky et al. 2010). The dog model has been the focus of many reports in the literature (Cabaud et al. 1981; Gupte et al. 2007; Krause et al. 1976; O’Connor 1976). Consequently, this model seems to have the highest amount of information available on identifying and comparing meniscal injuries in dog to human. The popularity of this model among meniscal investigators has been linked to the frequent veterinary hospital presentations and the ease of postoperative handling due to animal compliance, especially in trained dogs (Arnoczky et al. 2010; Luther et al. 2007). However, the use of this model is beginning to decrease due to the increasing pressure from animal interest groups in society (Webster et al. 2010). The sheep model, on the other hand, is getting more attention as a potential model for meniscal research (Arnoczky et al. 2010; Chiari et al. 2006; Kon et al. 2008; Kon et al. 2012). This could possibly be attributed to the mentioned pressure from animal interest groups opposing the use of dogs in research and the common use of sheep as a source for commercial meat production. The pig model is another practical and economically feasible model, commonly utilized in studies investigating meniscal biomechanics, ultrastructure, transplantation and repair techniques (Aspden et al. 1985; Ghadially et al. 1986; Ikeuchi et al. 1998; Jiang et al. 2012). The cow meniscus has been utilized in a limited number of reports in the literature; however, based on anatomical measurements the cow meniscus was found to be significantly bigger than the human meniscus (Proffen et al. 2012) and thus the bovine meniscus will not be considered in this work.

The non-uniform shape and complex geometry of the meniscus requires the availability of detailed knowledge of meniscus size and dimensions in human and other species commonly used in meniscal studies. Although anatomical features are only part of the criteria that are used before choosing an appropriate animal model for meniscal investigations, the availability of such anatomical information will be useful to investigators in different realms of science. Moreover, they can serve as a guide or a baseline to build upon in designing studies that depend on the geometry of the meniscus. For instance, meniscal transplantation is a promising and emerging procedure that provides an alternative treatment option to patients who have had total meniscectomy (Shelton 2007). The advantages and potential benefits of meniscal transplantation have been proposed and supported by many clinical studies (Cameron and Saha 1997; Goble et al. 1999; Paletta et al. 1997; Shelton and Dukes 1994; Siegel and Roberts 1993) and experimental studies performed in animal models (Aagaard et al. 1999; Cook et al. 2006; Lazovic 1998; Lazovic et al. 1997a; Lazovic et al. 1997b; McNickle et al. 2009; Rijk and Van Noorden 2002; von Lewinski et al. 2008). However, the existing difficulty in obtaining fresh grafts due to the limited number of human donors and the need for size matching between the donor and the recipient demand the development of an effective preservation and storage method that can be used to bank donor meniscal tissue. Vitrification or ice-free cryopreservation is a promising tissue preservation technique that can provide indefinite storage of orthopaedic tissues while maintaining their structural properties and cellular viability as was recently demonstrated for intact human knee articular cartilage (Jomha et al. 2012; Jomha et al. 2014). Tissue vitrification requires high concentrations of cryoprotectant agents (CPAs) and rapid cooling to achieve an amorphous glassy state (Fahy et al. 1983). CPA permeation kinetics (Abazari et al. 2012a; Abazari et al. 2009; Abazari et al. 2012b; Jomha et al. 2009; Yu et al. 2013) need to be mathematically modeled (Lawson et al. 2012) to minimize CPA exposure and subsequent risk of cellular toxicity. Because of the effect of tissue dimensions on permeation kinetics, anatomical dimensions will be a critical input to the mathematical models for meniscal cryopreservation development.

To date, there is little data on meniscus volume, weight and physical dimensions. If there were sufficient data, it could be used to select an appropriate animal model to advance meniscal surgical repair, or in mathematical models to advance meniscal preservation and transplantation. In major textbooks and reports in the literature, the anatomical description of the meniscus is limited to the general appearance, vascularity pattern and attachments (Arnoczky 1992; McDermott et al. 2010; Kummer 1987; Makris et al. 2011). In an anatomical study (Proffen et al. 2012) that compared the human knee and its structures with the knees of six animal species: pig, sheep, cow, goat, rabbit and dog, the dimensions of the meniscus were determined in all species but were limited only to the width and anterior–posterior length. The authors (Proffen et al. 2012) found that the sheep and goat stifle joints had the closest resemblance to the human knee and that these would likely make good models for meniscal research. Based on such findings, and given that our laboratory had used the pig stifle joint as a model for musculoskeletal research, we performed a more detailed and extensive anatomical study comparing the human menisci with those of sheep and pig. Therefore, the purpose of the current study was to compare the wet weight, volume and detailed dimensions of the body of the human medial and lateral menisci with those of sheep and pig menisci. Comparison of the size and dimensions is focused on the meniscal body, the area extending between the anterior and posterior horns.

## Methods

### Tissue collection

Six medial and six lateral healthy menisci (3 paired and 3 unpaired sets of two) were harvested from refrigerated knees of 9 deceased human male donors with an average age of 47 (range, 20 to 61 years), sourced from a tissue banking facility (Comprehensive Tissue Centre, Edmonton, Alberta, Canada). Approval of the Research Ethics Board of the University of Alberta, Edmonton, Canada was obtained and institutional safety and ethical guidelines were followed. All specimens came from deceased, unidentified organ and tissue donors. When both menisci were taken from the same joint (*n* = 3), they were considered as separate samples not as a pair of samples, unless indicated in the study. Sheep menisci (*n* = 6 medial and lateral) were obtained from adult Columbia and Clun Forest sheep (SunGold Specialty Meats Ltd., Innisfail, Alberta, Canada). Pig menisci (*n* = 22 medial and lateral) were obtained from adult Yorkshire pigs (Parkland Packers, Stony Plain, Alberta, Canada). All animal joints used in this study were from the hind leg (stifle joint) of sexually mature, male and female animals that were sacrificed for commercial meat consumption at two local abattoirs. However, the age, weight range or exact gender of the animal could not be determined due to the sources of the tissue. Following death, all animal joints were freed from skin and attached muscles, sectioned from mid-thigh to the lower third of the hind leg, placed in plastic bags and refrigerated for up to 48 h until dissected.

### Tissue dissection

Human knee joints were dissected by cutting the collateral ligaments to expose the joint followed by careful removal of the cruciate ligaments and adherent joint capsule. Tissue hydration was maintained with phosphate buffered saline (PBS) irrigation of exposed surfaces inside the joint to prevent water evaporation. Only menisci with no signs of injury or degenerative changes either to the meniscus or the underlying articular cartilage were included in the study. Intact medial and lateral menisci were removed en bloc and were further freed from synovial attachments, washed in PBS to remove excess synovial fluid, patted dry with sterile gauze and weighed using a standard scale.

In stifle joints, the dissection started anteriorly with a supra-patellar incision that was extended inferiorly on both sides to remove the patella, patellar tendon and surrounding fat pad. The collateral ligaments on both sides were excised to expose the periphery of both menisci. Removal of the patella allowed for clear visualization of the anterior joint structures and provided stability when the joint was turned over to expose the posterior joint structures. Then, the dissection was carried out posteriorly to remove the popliteal blood vessels, posterior joint capsule and all adherent soft tissue. Subsequently, the posterior horn of the lateral meniscus was carefully excised from the base of its bony attachment to the posteromedial corner of the medial femoral condyle and the posterior cruciate ligament (PCL) was severed from its posterior tibial insertion site(s). Next, stifle joints were put into flexion and the anterior cruciate ligament (ACL) was severed either from the base of its anterior tibial insertion or mid-way along its course between the femur and tibia. At this point, the femoral condyles were separated from the rest of the joint to provide a clear top view of the menisci. Finally, cutting from the peripheral border to the anterior and then posterior, each meniscus was separated from the remaining adherent membrane and the horns excised at the bony attachments followed by complete removal of the menisci. Intact medial and lateral menisci were washed in PBS to remove excess synovial fluid. Menisci were then patted dry and weighed prior to measurements.

### Volume determination

Volume displacement was performed based on Archimedes principle. Each medial and lateral meniscus included in the study was fully immersed in a 25 mL calibrated glass cylinder, graded with 0.5 mL increments, containing 15 mL of PBS, ensuring that the upper edge of the meniscus was sitting below the fluid line. The difference in volume before and after placement was attributed to the total volume of the body of the meniscus with the horns. Herein, the meniscal horns were included in the volume determination to allow for comparison with similar findings in the literature.

### Meniscal body dimension measurements

**Fig. 1:**
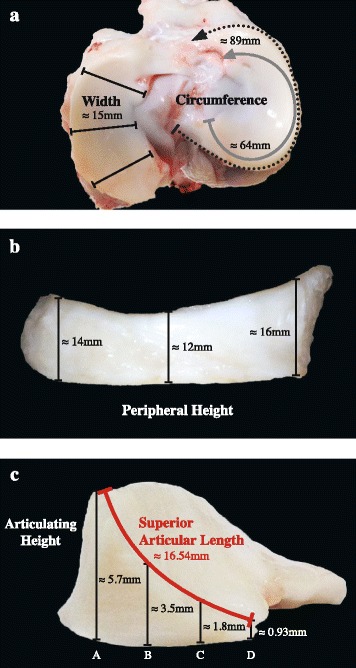
Meniscal dimensional measurements in a pig stifle joint representative of all measurements recorded across species. Panel **a** shows the two circumference measurements along the periphery of the meniscal body (*grey solid semi-circular line*) and the whole periphery (*dotted black semi-circular line*), and the width of the meniscal body as measured from the three locations, indicated with *black solid lines*. Panel **b** shows the three locations for the peripheral height, arranged from right to left: anterior, middle and posterior third. Panel **c** shows a cross-sectional view at the mid-point in a pig meniscus demonstrating the superior articular length (*red solid curved line*) and the articulating height (*black vertical lines*) at the four measurement points (A, B, C and D)

Numerous dimensions were measured directly on the surface of each meniscus. Dimensions included circumference, width, peripheral height, height of the articulating surfaces from base to top, and the length of the superior articular surface that accommodates the femoral condyle (Fig. 1). Two circumference measurements were quantified using a flexible, plastic tape measure that was molded: the first was around the peripheral rim of the body of each meniscus from the junction of the fiber bundles of the anterior horn with the body to the posterior horn junction with the body; the second circumferential measurement included the body and both horns, from the tip of the anterior horn to the end of the posterior horn (Fig. 1a). A plastic Vernier caliper was used to measure width and peripheral height of the body of both medial and lateral menisci at three different locations, moving from the front to the back: anterior third, middle third and posterior third. Width was recorded horizontally from the outer border to the innermost border (Fig. 1a). Peripheral height was measured vertically at the periphery from base to top (Fig. 1b). Length of the concave, sloped curvature of the superior articular surface facing the femoral condyle was measured with a tape measure. The tape measure was contoured from the tip of the highest point (peripherally) to the lowest point of the innermost edge (Fig. 1c) at three locations for each meniscus: anterior, middle and posterior. Height of the articulating surface, which represents a vertical measurement of the mass of the meniscus located between the two opposing articular surfaces (the upper one facing the femoral condyle and the bottom one that is sitting on the tibial plateau) was then measured. This measurement was recorded only in the middle third of the body, at four equally distanced points: a, b, c and d, starting from a point that was 2 mm in from the peripheral border until the innermost thin edge (Fig. 1c). Here, one jaw of the Vernier caliper was placed on the upper femoral surface while the other jaw was placed below the flat bottom surface. Both jaws were moved in a stepwise manner to the four locations at equally calculated distances.

### Statistical analysis

Descriptive and comparative statistical analysis was performed to determine differences between species using one-way ANOVA with Bonferroni’s post-hoc testing. *P*-values < 0.05 were considered statistically significant. Values are reported as mean ± standard error of the mean (SEM). Additionally, all dimensional parameters went through further analysis to determine the difference between lateral and medial menisci within each of the study species using one-way ANOVA with Bonferroni’s post-hoc testing. All statistical tests were performed using SPSS version 21.0 (IBM Corp. Released 2012. IBM SPSS Statistics for Windows, Version 21.0. Armonk, NY, USA: IBM Corp).

## Results

### Observations

**Fig. 2:**
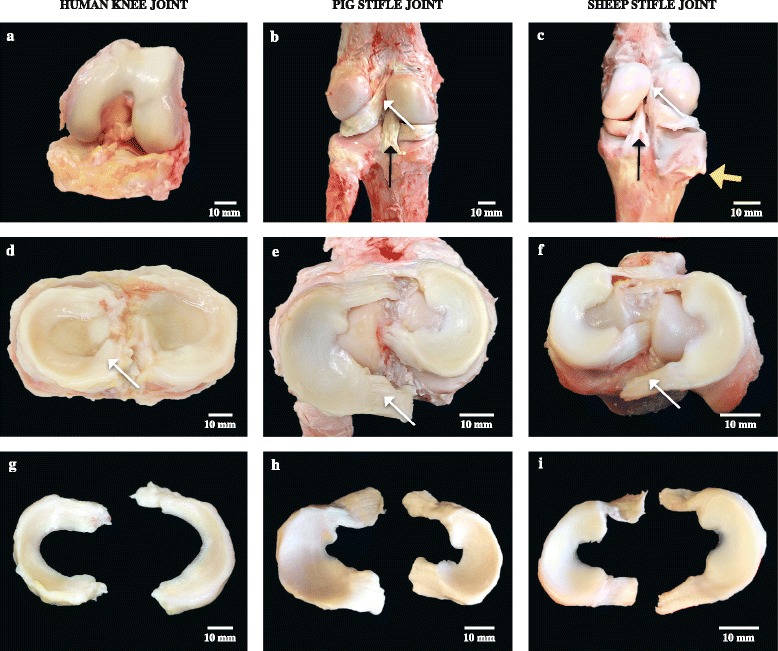
Different views of a human knee and animal stifle joints arranged into three columns and rows. *Top row*: **a** shows an anterior view of a partially opened left human knee joint after severing the anterior cruciate ligament; **b** and **c** posterior views of left pig and right sheep stifle joints, showing the femoral and tibial parts. This back view demonstrates the attachment site of the posterior horn of the lateral meniscus into the postero–lateral corner of the medial femoral condyle (*thin white arrows*) and below there is the posterior cruciate ligament (PCL) insertion site into the tibia (*vertical black arrows*). Noticeable is the absence of the fibula in the sheep joint (*thick yellow arrow*). *Middle row*: **d**, **e** and **f** showing top views of the medial and lateral menisci with their tibial insertion sites after removal of the femoral condyles and cutting the cruciate ligaments. Medial meniscus horns attach to the tibial plateau in both human and animal joints. However, in contrast to the human lateral meniscus, only the anterior horn of the lateral meniscus in pig and sheep attaches to the tibial plateau. The posterior horn of the lateral meniscus (*white arrows* in panels **e** and **f**) is hanging free after being separated from its insertion to the medial femoral condyle. The anterior horn of the medial meniscus was the most anterior structure across species. *Bottom row*: shows the morphology of the medial and lateral menisci after complete separation from the joint surface. **g** Left, human lateral meniscus with symmetrical width from front to back. Right, medial meniscus, which widens gradually towards the back; **h** the pig lateral meniscus is to the left and the medial meniscus is to the right; **i** the sheep medial meniscus is to the left and the lateral meniscus is to the right. The scale bars are approximations based on the average measurements of meniscal dimensions as reported in Tables 2, 3 and 4

All observations presented herein describe major differences noticed visually during dissection of animal joints as they relate to the human knee with emphasis on the overall shape of the meniscal body in all species. Photographs comparing the human joint and menisci to those from pig and sheep are shown in Fig. 2.

The body of both medial and lateral menisci looked smooth and glistening. In pig and sheep, the body of the medial meniscus was more tightly attached to the capsule throughout its peripheral border leading to restricted mobility, while the body of the lateral meniscus was slightly more mobile with a small area located at the middle third that was devoid of capsular attachment, permitting the tip of the scalpel to be passed underneath it. The body of the lateral meniscus had a more uniform width from front to back, while the body of the medial meniscus became wider toward the back. The medial meniscal body in both animals had a noticeable curve towards the back making it have a boomerang-like shape at the posterior third. The pig meniscal body was subjectively stiffer than human and sheep meniscal bodies. Moreover, the lateral meniscal body in pig had a small, notched protrusion at the junction of the body with the posterior horn.

**Fig. 3:**
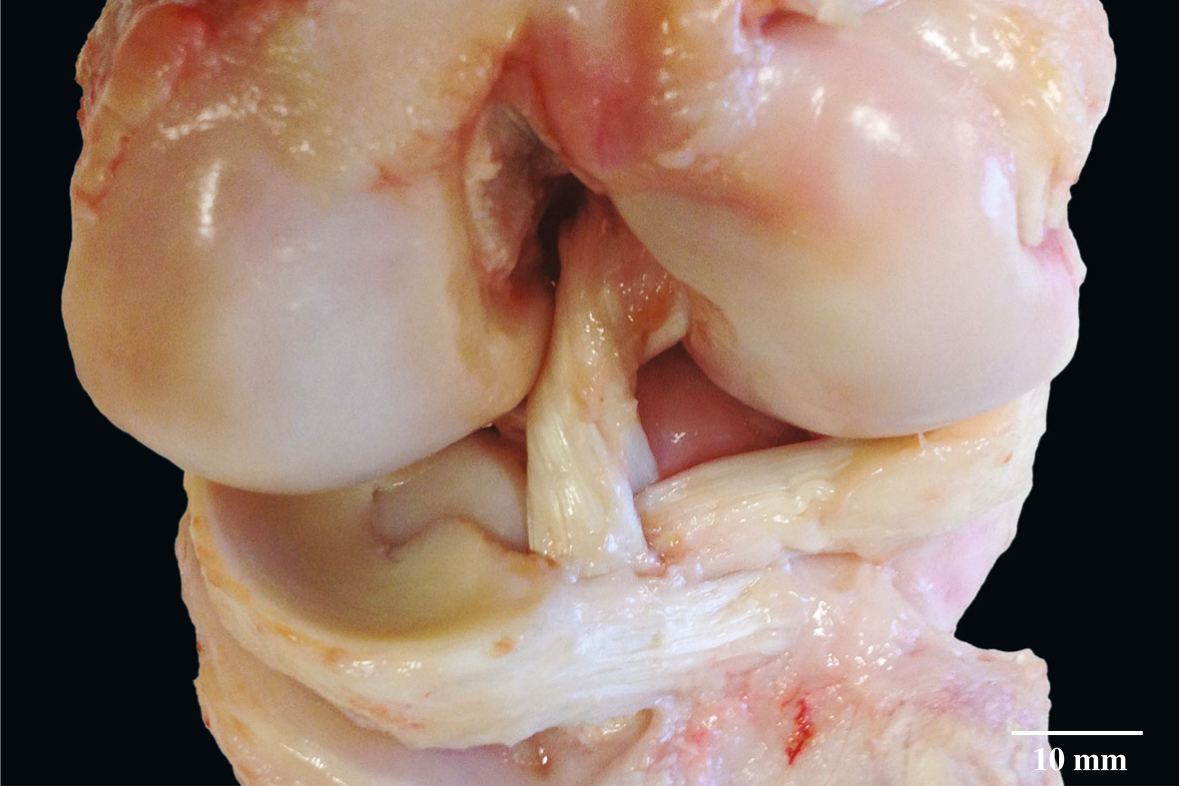
Pig joint with the two bundles of the anterior cruciate ligament (ACL) separated by the anterior horn of the lateral meniscus. The scale bar is an approximation based on the average measurements of meniscal dimensions as reported in Tables 2, 3 and 4

The horns in both sheep and pig were loose and more mobile than the body of the meniscus with visible penetrating blood vessels. Grossly, the horns were arranged in small bundles, in a fashion resembling the cruciate ligaments, running horizontally from the body to their insertion sites on the tibial plateau with thin layers of transparent septa surrounding the bundles. The anterior horn of the lateral meniscus separated the two bundles of the anterior cruciate ligament, which lay side by side in the sheep and pig joints (Fig. 3). In human knees the anterior and posterior horns of the lateral meniscus are attached centrally at the anterior and posterior intercondylar fossae respectively, while the horns of the medial meniscus had broader insertion sites on the edge of the tibial plateau. Sheep and pig anterior horn attachments on the tibial plateau were broader, away from the center, and the posterior horn of the lateral menisci passed behind the PCL to attach to the lateral aspect of the medial femoral condyle posteriorly. The sheep PCL had a wide posterior tibial insertion such that it almost looked as if it had two insertion sites.

### Direct measurements

Values presented include measurements taken from refrigerated (4 °C) medial and lateral menisci in human (*n* = 6), sheep (*n* = 6) and pig (*n* = 22).

### Weight and volume

Meniscal wet weights and volumes are presented in Table 1. The size of the meniscus varied among the species included in the study. The average wet weight and volume of human menisci were larger than sheep (at a ratio of sheep:human of 0.5:1 for both medial and lateral meniscus) and smaller than pig menisci at a ratio of pig:human of 1.3:1 for the lateral and 1.2:1 for the medial menisci (Table 1). Compared to human meniscus, the wet weight and volume of lateral menisci in both animals showed statistically significant differences, whereas for the medial menisci only the sheep medial meniscus had average weights and volumes that were significantly different (*p* = 0.004 and *p* = 0.007 for sheep medial meniscal weight and volume, respectively) from human meniscus. Across species, the lateral meniscus had greater weight and volume than the medial meniscus from the same species. In addition, there were strong correlations between the meniscal weight and volume within each group with a correlation coefficient (*R* ^*2*^) of 0.97 and 0.93 for the human medial and lateral menisci, respectively; for pig *R* ^*2*^ = 0.92 and 0.90 for the medial and lateral menisci, respectively; and for sheep *R* ^*2*^ = 0.96 and 0.76 for the medial and lateral menisci, respectively.

**Table 1:** The average meniscal wet weight (g) and volume (ml) in human, sheep and pig

	Mean ± Standard Error of the Mean (SEM)			
	Lateral meniscus		Medial meniscus	
	Weight (g)	Volume (ml)	Weight (g)	Volume (ml)
Human (*n* = 6)	**4.95 (±0.44)**	**5 (±0.53)**	**4.43 (±0.46)**	**4.5 (±0.58)**
Sheep (*n* = 6)	2.5 (±0.19)**	2.33 (±0.17)**	2.21 (±0.22)*	2.22 (±0.22)*
Pig (*n* = 22)	6.44 (±0.26)*	6.52 (±0.27)*	5.02 (±0.17)	5.09 (±0.18)

Human values are in bold. Statistically significant differences to the human menisci are indicated with asterisks, where (*) indicates significance levels with *p*-values < 0.05 and (**) indicates values that had a significance level with *p*-values < 0.001. Pig menisci had the largest values while the human menisci were larger than the sheep and smaller than the pig menisci

### Lateral meniscal body dimensions

Measurement and statistical significance of the results of the sheep and pig in comparison to the human lateral meniscus are summarized in Table 2. Dimensions of the lateral meniscal body were generally largest in pig menisci, with the exception of the circumference measurements. Human lateral menisci had the longest circumference for both the body and the whole circumference, which included the horns. Statistically significant differences from the human circumference were found in sheep (for the body and body with horns, *p* < 0.0005); in pig only the circumference of the body was significantly different (*p* < 0.0005). The whole circumference in the pig lateral meniscus was not significantly different (*p* = 0.370) from that of the human lateral meniscus, and that could be attributed to the longer horns in pig. Generally, for all dimensions other than circumference the values of the dimensions of the sheep lateral meniscus were not statistically different from those of the human. Whereas, the pig lateral meniscus, with the exception of the circumference, had dimensions that were significantly larger than the human lateral meniscus with an average measurement ratio of pig:human of 1.4:1 (range, 0.8–2.0). Comparing measurements taken from the three specified locations within every species, the horizontal width and peripheral height remained fairly consistent in human from front to back, a feature that was different from the animal menisci. In contrast to the human meniscus, the width and peripheral height of the lateral meniscus in the sheep and the pig were fairly similar in the anterior and posterior thirds while they always decreased in the middle third. Similarly, the sloped superior articular length in human lateral meniscus was greatest in the anterior third while it was greatest in the posterior third of pig and sheep lateral meniscus. The articulating height in all species, which was a parallel continuation to the peripheral height taken at the middle third, decreased gradually toward the innermost edge.

**Table 2:** Dimensions, in millimeters, of the lateral meniscus in the human, sheep and pig

	Lateral meniscus		
	Mean ± Standard Error of the Mean (SEM)		
	Human (*n* = 6)	Sheep (*n* = 6)	Pig (*n* = 22)
Circumference			
Body	**84 (±1.73)**	50.5 (±0.96)**	66.77 (±0.80) **
Body with horns	**103.5 (±1.6)**	73.83 (±2.27)**	94.54 (±2.17)
Width			
Anterior	**11.5 (±0.43)**	11.67 (±0.33)	15.95 (±0.23)**
Middle	**11.62 (±0.53)**	9.83 (±0.31)	14.63 (±0.35)**
Posterior	**11.67 (±0.33)**	11.33 (±0.42)	16.63 (±0.30)**
Average	**11.59 (±0.24)**	10.94 (±0.27)	15.74 (±0.2)**
Peripheral height			
Anterior	**6.41 (±0.89)**	7.08 (±0.49)	11.23 (±0.28)**
Middle	**6.33 (±0.49)**	4.83 (±0.31)	8.04 (±0.30)*
Posterior	**6.17 (±0.75)**	8.5 (±0.22)	13.13 (±0.29)**
Average	**6.3 (±0.39)**	6.8 (±0.41)	10.80 (±0.31)**
Articulating height			
Point - a	**4.0 (±0.45)**	3.25 (±0.17)	5.72 (±0.16)*
Point - b	**2.5 (±0.22)**	1.67 (±0.21)	3.5 (±0.17)*
Point - c	**1.13 (±0.13)**	0.93 (±0.03)	1.82 (±0.12)*
Point - d	**0.67 (±0.03)**	0.55 (±0.05)	0.93 (±0.02)**
Superior articular length			
Anterior	**13.5 (±0.43)**	10.67 (±0.42)	16.54 (±0.37)**
Middle	**12.5 (±0.72)**	10.33 (±0.49)	16.91 (±0.39)**
Posterior	**12.5 (±0.43)**	13.0 (±0.36)	19.45 (±0.31)**
Average	**12.83 (±0.32)**	11.33 (±0.37)	17.63 (±0.26)**

Human dimensions are in bold. Statistically significant differences from the human lateral meniscus are marked with asterisks

(*) indicates significance levels with *p*-values < 0.05 and (**) indicates significance level with *p*-values < 0.001

### Medial meniscal body dimensions

Medial meniscus dimensions and statistical significance in comparison to the human medial meniscus are summarized in Table 3. The circumference of the human medial meniscus was the largest and was significantly different from that of the sheep and the pig, for both measurements (*p* < 0.0005 for sheep and *p* = 0.042 for pig). For the remaining dimensions quantified, the sheep medial meniscus for the most part, was not significantly different from the human (*p* > 0.1); while different dimensions from the pig medial meniscus were significantly larger (*p* < 0.001) than the human medial meniscus with an average measurement ratio of pig:human of 1.3:1. The width in all species increased gradually from anterior to posterior. Sheep meniscal body width showed no statistically significant difference from the human meniscus other than the measurement at the posterior third of the sheep medial meniscus, which was significantly different from the human counterpart (*p* < 0.0005). The greater width of the pig medial meniscus was significantly different from the human meniscus except for the posterior third, which showed no statistically significant difference from the human meniscus at the same location. In contrast with the human medial meniscus, the sheep and the pig medial menisci had a peripheral height that was almost identical at the anterior and posterior thirds, and greater than the middle third measurement. The length of the superior articular surface was greatest in the posterior third of the medial meniscus in all species. The articulating height was found to decrease gradually towards the inner edge across all species.

**Table 3:** Dimensions, in millimeters, of the medial meniscus in the human, sheep and pig

	Medial meniscus		
	Mean ± Standard Error of the Mean (SEM)		
	Human (*n* = 6)	Sheep (*n* = 6)	Pig (*n* = 22)
Circumference			
Body	**88.67 (±2.13)**	55.5 (±1.33)**	64.95 (±1.04)**
Body with horns	**101.17 (±3.15)**	71.83 (±1.74)**	89.04 (±1.86)**
Width			
Anterior	**8.5 (±0.62)**	9.83 (±0.30)	14.23 (±0.32)**
Middle	**8.33 (±0.49)**	7.67 (±0.49)	12.23 (±0.30)**
Posterior	**14.83 (±0.79)**	10.83 (±0.48)**	16.00 (±0.37)
Average	**10.55 (±0.81)**	9.44 (±0.39)	14.15 (±0.27)**
Peripheral height			
Anterior	**5.5 (±0.34)**	5.83 (±0.31)	10.18 (±0.34)**
Middle	**5.0 (±0.45)**	4.42 (±0.37)	6.23 (±0.22)
Posterior	**7.0 (±0.68)**	5.83 (±0.47)	10.04 (±0.37)*
Average	**5.83 (±0.34)**	5.36 (±0.27)	8.82 (±0.29)**
Articulating height			
Point - a	**3.0 (±0.00)**	3.25 (±0.36)	4.45 (±0.14)**
Point - b	**1.58 (±0.20)**	1.5 (±0.34)	2.45 (±0.14)*
Point - c	**0.95 (±0.02)**	0.86 (±0.05)	1.13 (±0.07)
Point - d	**0.72 (±0.04)**	0.55 (±0.05)*	0.77 (±0.03)
Superior articular length			
Anterior	**9.67 (±0.80)**	10.5 (±0.72)	13.91 (±0.36)**
Middle	**9.15 (±1.8)**	10.33 (±0.49)	13.77 (±0.37)**
Posterior	**15.67 (±0.84)**	12.0 (±0.58)*	17.59 (±0.43)
Average	**11.49 (±0.98)**	10.94 (±0.37)	15.09 (±0.31)**

Human dimensions are in bold.

Statistically significant differences from the human lateral meniscus are marked with asterisks

(*) indicates significance levels with *p*-values < 0.05 and (**) indicates significance level with *p*-values < 0.001

### Lateral versus medial menisci

Table 4 shows a comparative summary and the significance level of the comparison between lateral and medial menisci within each group. There were no statistically significant differences found between lateral and medial menisci (*p* > 0.299) within human and sheep meniscal groups. However, pig menisci showed statistically significant (*p* < 0.001) differences between lateral and medial menisci across all dimensions except for the circumference which showed no difference (*p* = 1.0 and 0.519 for the body and body with horns, respectively).

**Table 4:** Comparison of lateral and medial meniscal dimensions within all three species

Lateral versus medial measurement comparisons (Mean ± SEM) and *p*-values									
	Human (*n* = 6)			Sheep (*n* = 6)			Pig (*n* = 22)		
	Lateral	Medial	*p*-value	Lateral	Medial	*p*-value	Lateral	Medial	*p*-value
Circumference									
Body	84 (±1.73)	88.67 (±2.1)	0.896	50.5 (±0.9)	55.5 (±1.33)	0.662	66.77 (±0.80)	64.95 (±1.04)	1.000
Body with horns	103.5 (±1.6)	101.1 (±3.1)	1.000	73.83 (±2.27)	71.83 (±1.7)	1.000	94.54 (±2.17)	89.04 (±1.86)	0.519
Width	11.59 (±0.24)	10.5 (±0.8)	1.000	10.94 (±0.27)	9.44 (±0.39)	0.357	**15.74 (±0.2)**	**14.15 (±0.27)**	**<0.0005**
Peripheral height	6.3 (±0.39)	5.8 (±0.3)	1.000	6.8 (±0.4)	5.36 (±0.27)	0.70	**10.80 (±0.31)**	**8.82 (±0.29)**	**<0.0005**
Articulating height									
a	4.0 (±0.45)	3.0 (±0.0)	0.299	3.25 (±0.17)	3.25 (±0.36)	1.000	**5.72 (±0.16)**	**4.45 (±0.14)**	**<0.0005**
b	2.5 (±0.22)	1.58 (±0.2)	0.406	1.67 (±0.21)	1.5 (±0.34)	1.000	**3.5 (±0.17)**	**2.45 (±0.14)**	**<0.0005**
c	1.13 (±0.13)	0.95 (±0.02)	1.000	0.93 (±0.03)	0.86 (±0.05)	1.000	**1.82 (±0.12)**	**1.13 (±0.07)**	**<0.0005**
d	0.67 (±0.03)	0.72 (±0.04)	1.000	0.55 (±0.05)	0.55 (±0.05)	1.000	**0.93 (±0.02)**	**0.77 (±0.03)**	**0.001**
Superior articular length	12.8 (±0.32)	11.49 (±0.9)	1.000	11.33 (±0.37)	10.94 (±0.37)	1.000	**17.63 (±0.26)**	**15.09 (±0.31)**	**<0.0005**

Here only the surface dimensions in millimeters are compared for human, sheep and pig menisci. *P*-values for the lateral versus medial meniscus comparisons are reported to the right of every major column. For the circumference, both measurements were included in the comparison. Whereas, for dimensions (width, peripheral height and superior articular length) that were recorded from three different topographical locations, only the representative average of the three measurements was included in the comparison. While for the articulating height which was recorded only from the middle third of every meniscus, measurements from all four points were compared. Statistically significant differences are indicated in bold. Aside from the circumference measurements, only the pig menisci showed significant differences between the lateral and medial menisci

## Discussion

The purpose of this descriptive laboratory study was to develop an understanding of the human meniscus weight, volume and anatomical dimensions, and to compare those parameters with sheep and pig menisci.

It was observed that both sheep and pig joints are grossly similar to their human counterpart but there are also differences. The data showed interspecies and intra-group variability in some of the measured parameters. Both animal models are cost-effective and readily available options that can provide joints to be used for scientific investigation. The average cost of each joint was $4.80 (Canadian dollars) for sheep and $3.30 (Canadian dollars) for pig. Subjectively, the observed large amount of anterior fat pad in pig joints could lead to difficulty with arthroscopic device insertion into the joint (Voto et al. 1988). Pig menisci demonstrated increased stiffness and the sheep meniscus was softer, making it easier to create surgical lesions for meniscal research using the sheep model (Cake et al. 2013).

For the direct dimensional measurements, the body of each meniscus was divided into three cross sectional locations: anterior third, middle third and posterior third. Dimensions were measured individually at these three topographical locations. Results are shown in tables summarizing individual measurements from each location and the average of all three locations was also included. Additionally, within each species, lateral versus medial dimensional differences are reported with the significance level. Circumference, width, peripheral height, articulating height and length of the superior articular surface were measured from the body of every meniscus included in the study. Data reported herein should help to establish an understanding of the anatomical measurements in the human menisci and to provide direct evidence that the sheep model, from a comparative anatomy point of view, is a suitable and readily-available model that can be used to evaluate meniscal investigations with the aim of applying findings to human meniscus.

Human menisci have been the focus of the majority of reports found in the literature. Most reports have looked specifically into certain, but not all, aspects related to studying the human meniscus anatomically. Magnetic resonance imaging (MRI) has been used to quantify meniscal sizing and dimensional parameters (Bowers et al. 2007; Stone et al. 1994). Our findings for the human meniscal volume differ from previously published studies that used similar volume determination techniques. In a study of 21 fresh frozen cadaver knees (Stone et al. 1994), meniscal volume was determined by using 3D MRI then each volume measurement was compared to its respective value that was determined by the water displacement method. The authors reported the water displacement volume as 2.5 ml (SEM, ± 0.3 ml) for the medial meniscus and 2.5 ml (SEM, ± 0.2 ml) for the lateral meniscus in human. Furthermore, the MRI technique was found to consistently underestimated the true volume of the meniscus (Stone et al. 1994). Another study (Bowers et al. 2007) also determined the meniscal volume of a human cadaver knee using MRI and compared each specimen to its respective water displacement volume. They reported mean volume of 3.04 ml (SD, ± 0.04 ml) for the medial menisci and 3.07 ml (SD, ± 0.07 ml) for the lateral menisci. In our study, we found the volume to be 4.5 ml (SD, ± 1.4 ml) and 5 ml (SD, ± 1.3 ml) for the medial and lateral menisci, respectively. Differences observed between our findings and previous reports could be attributed to our use of refrigerated, male menisci while the authors from the two previous studies (Stone et al. 1994) and (Bowers et al. 2007) used frozen samples; freezing is known to cause tissue dehydration, leading to a post-thawing volume that may be lower than the initial volume. Moreover, in our study adequate tissue hydration was maintained throughout the dissection process by irrigating exposed surfaces inside the joint with PBS to decrease water evaporation during dissection, which was not mentioned in the other studies. Perhaps more importantly, the genders of the cadaver menisci, which was not reported in the two previous studies (Stone et al. 1994) and (Bowers et al. 2007), could have been another contributing factor. This factor could also explain the observed differences as we used only male menisci in our study.

Dimensional parameters similar to those reported in this study for human menisci were found in a small number of studies (Bloecker et al. 2012; Erbagci et al. 2004; Hunter et al. 2006; McDermott et al. 2004; Shaffer et al. 2000; Stone et al. 2007; Wirth et al. 2010; Yoon et al. 2011a; Yoon et al. 2011b). In some of those studies, despite the complexity of their experimental protocols, the dimensional measurements did not focus on the body of the meniscus that has the greatest contribution to the knee mechanics. The width, height and anterior-posterior length were the most commonly investigated dimensional parameters. Moreover, the terminology used could be confusing. For instance, the width of the meniscus was usually measured from the peripheral edge to a line connecting the anterior and posterior horns while the menisci were still attached to the tibial plateau (Shaffer et al. 2000; Stone et al. 2007; Yoon et al. 2011a; Yoon et al. 2011b). This width does not describe the portion of the meniscus itself that undertakes the biomechanical functions. Instead, it also includes the area of the tibial plateau enclosed by the meniscus, extending from the most peripheral border of the meniscus at the meniscosynovial junction to the horns insertion. This is a more clinical approach to width as opposed to our more anatomical approach. In this study, we measured the width of the body of human menisci at three different locations: anterior third, middle third and posterior third to account for the widening observed in the medial meniscus from front to back, and determined the average of the three measurements. Erbagci et al. (2004) in a dimensional MRI study of normal menisci in 174 healthy male and female subjects (mean age 29, range 18–60) reported the mid-body width at 7.4 mm (SD, ± 2.65 mm) and 8.4 mm (SD, ± 0.83 mm) for the medial and lateral menisci, respectively. McDermott et al. (2004) in an anatomical cadaveric study of meniscal allograft sizing, used a digital Vernier caliper to determine the width of meniscal body at the mid-portion of 44 lateral and 44 medial menisci. They reported a meniscal body width of 9.3 mm (SD, ± 1.3 mm) and 10.9 mm (SD, ±1.3 mm) for the medial and lateral menisci, respectively. In our study, we found the width at the middle third of human menisci to be 8.3 mm (SD, ± 1.2 mm) for the medial meniscus and 11.6 mm (SD, ± 1.3 mm) for the lateral meniscus. Bloecker et al. (2012) and Wirth et al. (2010) used 3-dimensional reconstruction of MRI images to measure meniscal width at different locations and reported only the average of all measurements. Wirth et al. (2010) studied intact medial menisci of 11 female subjects (mean age 55.3 ± 6.0 years) and reported the mean width of the female medial meniscus as 8.96 mm (SD, ± 0.5 mm), while Bloecker et al. (2012) who studied healthy medial and lateral menisci of 47 male subjects (mean age 57 ± 9 years, range 45–79) reported the mean width as 9.92 mm (SD, ± 1.0 mm) for the medial meniscus and 10.1 mm (SD, ± 1.2 mm) for the lateral meniscus. We found that the mean width in male menisci was 10.55 mm (SD, ± 3.4 mm) and 11.59 mm (SD, ± 1.0 mm) for the medial and lateral menisci, respectively. These differences suggest the need for gender stratification of meniscal dimensional measurements.

The peripheral height of intact human meniscal body, also termed “maximal thickness” by some authors (Bloecker et al. 2012; Wirth et al. 2010), was found in a limited number of studies. Erbagci et al. (2004) reported the height at mid-body as 5.03 mm (SD, ± 0.91 mm) for the medial meniscus and 4.94 mm (SD, ± 0.9 mm) for the lateral meniscus. Wirth et al. (2010) determined this measurement at the highest point of the medial meniscal body, which they termed maximal thickness, in 11 female subjects and found it to be 6.72 mm (SD, ± 1.45 mm). Bloecker et al. (2012) reported the height, also termed as maximal thickness, as 7.7 mm (SD, ± 1.13 mm) and 7.2 mm (SD, ± 0.97 mm) for the medial and lateral meniscus, respectively. Values consistent with these are reported herein, and we found that the peripheral height was greatest posteriorly with a value of 7.0 mm (SD, ± 1.6 mm) and 6.2 mm (SD, ± 1.8 mm) for the medial and lateral menisci, respectively. Interestingly, contrary to healthy menisci, the height of menisci from 257 symptomatic osteoarthritic females was found to be lower with an average value of 2.9 mm (SD, ± 2.0 mm) for the medial meniscus and 5.4 mm (SD, ± 2.5 mm) for the lateral meniscus (Hunter et al. 2006). The latter suggest an alteration in the meniscal dimension due to osteoarthritic changes and stress the importance of utilizing healthy meniscal tissue.

The ability of the meniscus to function as a load distributor is dependent on the robustness of the peripheral border to withstand axial loading and convert it into circumferential hoop stress within the collagen fibers (Makris et al. 2011). The circumference length has been quantified by Kohn and Moreno (1995) and McDermott et al. (2004). Kohn and Moreno (1995) in a cadaveric study used a non-elastic polyester thread that was placed along the periphery of 56 medial and lateral human menisci, including their horn insertions. They stratified menisci into left knee and right knee groups. Their report showed a peripheral length of 110.86 mm (SD, ± 13.18 mm) and 111.15 mm (SD, ± 11.07 mm) for the medial and lateral menisci of the right knee joints; and there was no significant difference between menisci taken from the right and left knees of the same donors. McDermott et al. (2004) in an anatomical study measured the circumference in 44 menisci using a thin steel wire that was molded around the peripheral rim of each meniscus from the anterior to the posterior bony insertion. They reported the circumference length as 99.0 mm (SD, ± 9.3 mm) and 91.7 mm (SD, ± 9.6 mm) for the medial and lateral menisci, respectively, noting that the medial meniscus was longer than the lateral meniscus. Those measurements are comparable to those reported in the current study as 101.17 mm (SD, ± 7.7 mm) and 103.5 mm (SD, ± 3.9 mm) for the medial meniscus and lateral meniscus, respectively. Additionally, we have included another measurement that was not limited to the whole peripheral length of the meniscus but excluded the horns resulting in a measurement of the meniscal body without the horns, which was not found in any report obtained.

Limitations in this study were the small sample sizes in the human and sheep groups, and the inclusion of only male donors in the human group. Although the latter helped in reducing the variability in the parameters measured, it precluded the determination of the gender-based differences in the human samples. However, the inclusion of only human male menisci was not selective and it was merely due to the supply and availability of deceased donor knees from a local tissue banking facility within the time-frame of the study. Another limitation was not measuring the antero-posterior (A-P) length of all menisci included in the study. However, measurements were performed after specimens were completely separated from the tibial plateau, a feature that could change the arc or the curve at which the menisci turn to insert into the tibial plateau contributing to inaccurate lengths, especially in the human lateral meniscus, which has more central anterior and posterior insertions. Nonetheless, in a small subset of six medial and lateral menisci in pig (not reported), this length was measured and was compared to the circumference of the respective sample. In the same subset, we found that the A-P length in pig menisci represented an average of 46.63% (range, 42.22–51.90) of the whole circumference in lateral menisci and 46.31% (range, 46.18–51.67) of the whole circumference in medial menisci. When compared with the circumference of the body alone, the percentage increased to 57.13% in the pig lateral meniscus and to 55.21% in the pig medial meniscus.

Despite the cross-species similarities in the shape of the knee and meniscus, quadruped knees are loaded in the flexed position, while human knees are fully loaded in extension indicating differences in the range of motion at which animal menisci perform their biomechanical functions (Gupte et al. 2007). Despite the positional difference, the sheep model was found to have biomechanical and histological features that would make it a more suitable model for meniscal studies (Chevrier et al. 2009; Sandmann et al. 2013). Biomechanically, Sandmann et al. (2013) compared several viscoelastic properties of cow, sheep and pig menisci to human menisci and reported that the ovine (sheep) model demonstrated the highest similarities to the human menisci (Sandmann et al. 2013). Moreover, Chevrier et al. (2009), using histology and scanning electron microscopy performed a study comparing the sheep and rabbit meniscus to the human meniscus. They reported the sheep meniscus to have greater structural similarity to the human meniscus in terms of vascularization patterns, cell density and extracellular matrix collagen ultrastructure. Burger et al. (2007) who investigated the effect of sutured and unsutured radial meniscal tears on the articular cartilage degeneration in 20 sheep, reported that their meniscus displayed a pattern of articular cartilage degeneration that was similar, although more rapid, to what has been reported (Cicuttini et al. 2002; Sommerlath 1991) for the human meniscus. In support of these reports, our study found that the human medial and lateral meniscal dimensions were most similar to the sheep medial and lateral menisci. However, the weight and volume of the human medial meniscus was more comparable only to the pig medial meniscus. Although medial and lateral menisci differ in their geometry, propensity to and behavior after injury, our findings showed that both medial and lateral menisci were statistically similar from an anatomical measurement point of view in both human and sheep. However, for pig menisci, there were significant differences in almost all the dimensions, corresponding with the differences in the geometry, weight and volume between medial and lateral porcine menisci.

## Conclusion

Evaluation and development of new treatment options or products to repair human meniscus requires testing in an appropriate animal model to allow for clinical translation. In this study, with respect to physical anatomy, we found that sheep lateral and medial meniscal dimensions more closely matched the human meniscal dimensions when compared to those of pig. Based on observational and dimensional features of the two animal models reported in this study, our data indicated that the main dimensional parameters of the sheep model would make it a more suitable animal proxy, supporting other reports advocating the use of the sheep model in meniscal research related to physical dimensions. As anatomical parameters are only part of the criteria used in selecting animal models, further investigations are required to determine the similarity between human and sheep menisci biomechanically and structurally.
